# Identification of Unique and Conserved Neutralizing Epitopes of Vestigial Esterase Domain in HA Protein of the H9N2 Subtype of Avian Influenza Virus

**DOI:** 10.3390/v14122739

**Published:** 2022-12-08

**Authors:** Xiangyu Huang, Guihu Yin, Yiqin Cai, Jianing Hu, Jingwen Huang, Qingtao Liu, Xiuli Feng

**Affiliations:** 1 Key Laboratory of Animal Microbiology of China’s Ministry of Agriculture, College of Veterinary Medicine, Nanjing Agricultural University, Nanjing 210095, China; 2 Key Laboratory of Veterinary Biological Engineering and Technology, Ministry of Agriculture, Institute of Veterinary Medicine, Jiangsu Academy of Agricultural Sciences, Nanjing 210014, China; 3 MOE Joint International Research Laboratory of Animal Health and Food Safety, College of Veterinary Medicine, Nanjing Agricultural University, Nanjing 210095, China

**Keywords:** H9N2 avian influenza virus, HA protein, neutralization, monoclonal antibody, antigen epitope

## Abstract

The H9N2 subtype of avian influenza virus (AIV) has been reported to infect not only birds, but also humans. The hemagglutinin (HA) protein is the main surface antigen of AIV and plays an important role in the viral infection. For treatment strategies and vaccine development, HA protein has been an important target for the development of broadly neutralizing antibodies against influenza A virus. To investigate the vital target determinant cluster in HA protein in this work, HA gene was cloned and expressed in the prokaryotic expression vector pET28a. The spleen lymphocytes from BALC/c mice immunized with the purified recombinant HA protein were fused with SP2/0 cells. After Hypoxanthine-Aminopterin-Thymidine (HAT) medium screening and indirect ELISA detection, six hybridoma cell lines producing anti-HA monoclonal antibodies were screened. The gradually truncated HA gene expression and western blotting were used to identify their major locations in epitopes specific to these monoclonal antibodies. It was found that the epitopes were located in three areas: ^112^NVENLEEL^119^, ^117^EELRSLFS^124^, and ^170^PIQDAQ^175^. Epitope ^112^NVENLEEL^119^ has a partial amino acid crossover with ^117^EELRSLFS^124^, which is located in the vestigial esterase domain “110-helix” of HA, and the monoclonal antibody recognizing these epitopes showed the neutralizing activity, suggesting that the region ^112^NVENLEELRSLFS^124^ might be a novel neutralizing epitope. The results of the homology analysis showed that these three epitopes were generally conserved in H9N2 subtype AIV, and will provide valuable insights into H9N2 vaccine design and improvement, as well as antibody-based therapies for treatment of H9N2 AIV infection.

## 1. Introduction

The H9N2 subtype of avian influenza is now common in many eurasian nations and can reduce chicken egg production or co-infect with other infections, resulting in substantial mortality and significant economic losses [1,2,3,4,5]. H9N2 subtype AIV was first isolated in turkeys in 1966 [6], and was first isolated and identified in 1992 in China [7]. Following this, H9N2 subtype AIV started to appear in additional regions [8,9,10]. The H9N2 subtype of AIV can harm avian physiology and weaken immune systems, leaving them more vulnerable to other harmful pathogenic microorganisms [10,11]. Furthermore, H9N2 AIV has been reported to infect humans [12,13,14,15] and can also pass on gene snippets or even full genomes to other highly deadly AIVs, including H4N6, H5N6, H5N2, H7N2, H10N8, and H7N9 [8,16,17,18,19,20].

AIV is a segmented negative-strand RNA virus, and eight viral segments encode the following proteins PB2, PB1, PA, HA, NP, NA, M, and NS. One of the most significant glycoproteins among them is the hemagglutinin HA, whose amino acids are subject to mutation [21,22,23,24,25,26]. As a result, the immune system may develop resistance to the vaccination due to antigenic drift and immunological escape. When HA, which enters cells by attaching to the sialic acid on the cell surface, is targeted, specific neutralizing antibodies (Abs) are generated [5,27]. The identified neutralizing antibodies primarily target five immunodominant antigenic sites that are extremely changeable on the structural domain of the HA globular head [28,29,30,31], preventing the adhesion or fusion mediated by HA. However, the neutralizing antibodies located in the head can only produce the neutralizing activity against one or a few strains and are not widely used [32]. Antibodies directed against the stem of the HA protein have also been investigated, which barely have the neutralizing activity [33]. Recently, a third class of antibodies has been identified: those directed against the vestigial esterase domain (VED) [34,35,36,37,38], which is very conserved in the same isotype and varies between isotypes [39,40,41]. Previous studies have reported on H9N2-associated antigenic epitopes. Zhu et al. [42] screened six important antigenic sites by monoclonal antibodies and their recognition of variant sites in different strains: 92, 145, 166, 167, 168, and 197. Wan et al. [43] found nine very important sites, in which 164, 167, 168, 196 and 207 were newly identified. Wang et al. [44] found rSNHA-200 produced considerably more neutralizing antibodies and provided the highest protection against both homologous and heterologous H9N2 AIVs. Zhu et al. [45] found 53, 274, 83 and 276 were found to be important and neutral sites for the HA protein of H5N1 AIV, all of which are located in the VED region. However, no neutralizing epitopes targeting the VED region have been identified in H9N2 subtype AIV. In this paper, to investigate the potential epitope of B cell in HA protein, HA gene was cloned and expressed to be used as immunogen to prepare the hybridoma cells. Also, the epitope and neutralization activities of the monoclonal antibodies produced by the hybridmoa cells were performed. Results showed that the monoclonal 1E11 and 2B3 were the neutralizing and targeting epitopes ^112^NVENLEEL^119^ and ^117^EELRSLFS^124^, which are located in the vestigial esterase domain (VED). These results suggested that the monoclonal antibodies against this region might act as the neutralizing antibodies against H9N2 subtype AIV.

## 2. Materials and Methods

### 2.1. Virus 

The H9N2 (A/chicken/Shandong/LY1/2017) strain was isolated and preserved in our laboratory [46], which was injected into a 9-day-old SPF chicken embryo allantoic cavity, and the allantoic fluid was collected and stored at −80 °C.

### 2.2. Cells

SP2/0 cells were cultured in RPMI-1640 medium (350-000-CL, Wisent Biotechnology (Nanjing, China) with 20% fetal bovine serum (FBS, FSP500, EXCEL, China), 1% penicillin and 100 µg/mL streptomycin at 37 °C and 5% CO_2_.

MDCK and A549 cells were cultured in RPMI-1640 medium (350-000-CL, Wisent Biotechnology (Nanjing, China) with 10% FBS (FSP500, EXCEL, China), 1% penicillin and 100 µg/mL streptomycin at 37 °C and 5% CO_2_.

### 2.3. HA Gene Cloning and Recombinant Protein Expression

**Figure 1:**
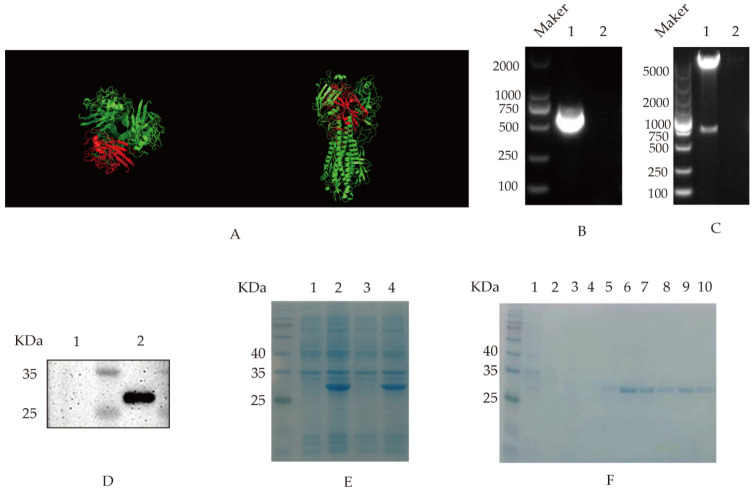
Selection and prokaryotic expression of the truncated HA genes: (**A**) the spatial structure of HA. The structure was predicted by the software SWISS-MODEL and analyzed by pymol software. The selected protein (HA:75AA–290AA) was marked in red. The left picture is an overhead view, and the right picture is a side view; (**B**) PCR amplification of HA. Lane 1 HA amplification; lane 2 negative control; (**C**) identification of recombinant plasmid pET-28a-HA with the double enzyme digestion. Lane 1, the recombinant plasmidpET-28a-HA; lane 2, pET-28a vector; (**D**) the recombinant HA protein reacted with the positive serum. The fusion protein pET-28a-HA was incubated with chicken-positive serum. Lane 1, pET-28a vector control; lane 2, pET-28a-HA (28KDa); (**E**) HA Protein expression identification. The recombinant HA protein was induced by IPTG and was analyzed on SDS-PAGE. Lane 1, pET-28a-HA not induced; lane 2, pET-28a-HA induced; lane 3, pET-28a-HA induced and supernatant after sonication; lane 4, pET-28a-HA induced and precipitated after sonication; and (**F**) purification of the recombinant HA protein. Lane 1, the effluent sample after column hanging; lanes 2–10, the effluent sample after elution with different imidazole concentrations, in which imidazole concentrations of 20, 30, 50, 80, 120, 150, 150, 2500 and 500 mM, respectively.

Based on the amino acid sequences of HA gene in GenBank (No. MH018677), the region (75–290AA) was selected for truncated expression based on the reported high-variance region of HA proteins prone to neutralizing antibodies [9] and the head of HA (75AA–290AA) predicted by software SWISS-MODEL (https://swissmodel.expasy.org/, accessed on 24 March 2021) and analyzed by software pymol 2.5 (Figure 1A). We extracted total RNA of influenza virus with Trizol and then performed RT-PCR: Oligo Dt Primer 1 μL; dNTP Mixture 1 μL; Total RNA 2 μL; Rnase Free dH_2_0 6 μL. Hold at 65 °C for 5 min and then cool rapidly on ice. The above denaturing reaction solution 10 μL; 5×PrimeScript II Buffer 4 μL; RNase Inhibitor 0.5 μL; PrimeScript II RTase 1 μL; RNase Free dH_2_O 4.5 μL. The reverse transcription reaction was carried out under the following conditions: 42 °C 60 min; 95 °C 5 min; 4 °C 10 min. The primers were designed to clone the truncated HA gene, using the following PCR cycles: the initial denaturation at 98 °C for 5 min, denaturation at 95 °C for 50 s, annealing at 58 °C for 30 s, extension at 72 °C for 40 s for 35 cycles and 72 °C extension for 10 min. The PCR product was purified by agarose gel electrophoresis and was cloned into plasmid pET-28a. The positive recombinant plasmid was identified by PCR and the double enzyme digestion identification with *Bam*H I and *Hind* III. Then, the recombinant plasmid pET-28a-HA was transformed into an *E. coli* BL21 (DE3) strain and was induced with 100 μM IPTG for 5 h, and the supernatant and precipitate samples of the induced expressed proteins were analyzed with sodium dodecyl sulfate-polyacrylamide gel electrophoresis (SDS-PAGE) and Western blotting. Next, the recombinant HA protein was purified by the nickel affinity chromatography, and the purified proteins were analyzed by SDS-PAGE.

### 2.4. Mice Immunization

Five 6~8-week-old female BALB/c mice were numbered from 1 to 3 and immunized with the purified recombinant HA protein emulsified with Freund’s complete adjuvant at a volume ratio of 1:1. Mice were intraperitoneally injected at a dose of 100 μg HA protein per mouse. The second and third immunizations were emulsified with Freund’s incomplete adjuvant, with an interval of two weeks between each immunization. At one week after the third immunization, the serum was isolated from the immunized mice, and the serum antibody levels were determined by indirect ELISA. The mouse with the highest antibody level was selected for booster immunization with 200 μg of the recombinant HA protein to prepare for cell fusion.

### 2.5. Cell Fusion and Identification of Monoclonal Antibodies

At 3rd day after the boosted immunization, the spleen cells were aseptically isolated and fused with SP2/0 cells following the action of PEG solution (P7171, SIGMA). The fused cells were cultured in HAT medium and screened by indirect ELISA. The positive hybridoma cells were subcloned three times to identify the specific hybridoma cell clone and were injected into BALB/c mice to prepare the ascites antibody. Then, the type and isotype of monoclonal antibodies were determined by a mouse monoclonal antibody isotype identification kit (PK20003, Proteintech). Finally, the specificities of mAbs were determined by western blotting and indirect immunofluorescence (IFA).

### 2.6. Enzyme-Linked Immunosorbent Assay (ELISA)

To screen the positive hybridoma cell clones, the ELISA diagnostic method was established using the HA recombinant protein as the encapsulated antigen. Wells of plates were coated overnight at 4 °C with 2 μg/mL of the purified HA protein diluted with carbonate–bicarbonate buffer (pH 9.6), and then were blocked with 300 μL PBST (0.01 M phosphate-buffered saline (PBS), pH 7.2, 0.05% Tween 20) containing 5% skim milk at 37 °C for 1 h. After washing, 100 μL hybridoma cell supernatant was added to each well of the plate and was incubated at 37 °C for 1 h. Next, 100 μL HRP conjugated goat anti-mouse IgG (H + L) antibody (BA1051, BOSTER BIOLOGICAL Technology) at a 1:5000 dilution was added to wells for 1 h at 37 °C. Then adding 100 μL TMB chromogen solution for 15 min at 37 °C (P0209, Beyotime Biotechnology, China) and adding 50 μL stop solution for TMB Substrate (P0215, Beyotime Biotechnology, China). The absorbance was measured at 450 nm using a microplate reader (Bio Tek).

The mouse monoclonal antibody isotype identification kit (PK20003, Proteintech) and the mouse monoclonal antibody subtype identification kit were removed from 4 °C and equilibrated at room temperature for 30 min. Ascites were diluted 1:100,000 in 1 x PBST. The sample to be tested was added to the sample wells of the slab at an appropriate dilution of 50 μL/well. Without incubation, add 1 x sheep anti-mouse IgA+IgM+IgG-HRP was added to the sample wells at 50 μL/well, mixed gently on a mixer or by tapping the sides of the plate holder for 1 min, covered with sealing film and incubated at room temperature for 1 h. The liquid was discarded from the wells and the plate was washed three times with 1×PBST and patted dry on blotting paper, and 100 μL/well of the ready-to-use colour development solution was added to the wells, along with 100 μL/well of termination solution to each well. The absorbance was measured at 450 nm using a microplate reader (Bio Tek).

### 2.7. Design and Expression of the Truncated HA Gene

To determine the B-cell epitope of these HA monoclonal antibodies, the truncated HA fragment was first split into two segments: HA-75 and HA-159. The truncated HA segments were cloned by PCR with primers containing *Bam*H I and *Sal* I enzyme site, and were cloned into a pET-28a(+) vector. After the recombinant protein was expressed, western blotting was performed with the screened mAb and His-tagged antibodies. To further localize the antigenic epitopes, the overlapping sequences of the HA protein of 100–181AA (HA-100) and 131–194AA (HA-131) were cloned with primers containing both *Bam*H I and *Sal* I enzyme sites, and connected into the prokaryotic expression vector pET-28a(+) vector. The recombinant plasmid was then transformed into *E. coli BL*21 (DE3) and was expressed. To finally confirm the antigenic epitopes, five polypeptides containing ten amino acids were synthesized step by step and were coupled to BSA carrier in Shanghai Apeptide Co., Ltd. (Shanghai, China). The antigenic determinant of mAb was finally identified by western blotting. The primers for the above HA truncated gene are shown in Table 1.

### 2.8. SDS-PAGE and Western Blotting

According to the experimental instructions, the prepared samples were added to the SDS-PAGE at 10 μL per well and were separated by SDS-PAGE (Shanghai Epizyme Biomedical Technology, China) with 12.5% and 15% in which the 15% protein gel was used mainly for epitope identification and the rest was used at 12.5%, then were transferred onto PVDF membranes (1620177, BIO-RAD, America). After blocking at room temperature for 2 h in 5% skim milk in PBST (0.01 M phosphate-buffered saline (PBS), pH 7.2, 0.05% Tween 20), the membranes containing protein samples were incubated overnight with a monoclonal antibody at 4 °C. After washing, the membranes were incubated with the HRP conjugated goat anti-mouse IgG (H + L) (BA1051, BOSTER BIOLOGICAL Technology) or a rabbit anti-chicken IgG/HRP (SE235, Solarbio) at 37 °C for 1 h each. Finally, the membranes were detected by a chemiluminescence detection kit (170–5061, BIO-RAD).

### 2.9. Indirect Immunofluorescence Assay (IFA)

MDCK cells were infected with or without MOI = 0.1 H9N2 subtype AIV for the indirect immunofluorescence with hybridoma cell supernatants. After 24 h, the MDCK cells were fixed with 4% paraformaldehyde for 10 min and were then incubated with 0.1% Triton-X for 10 min. After washing, the cells were incubated with hybridoma cell supernatant or anti-NP mAb, which was provided by in-house preparation [47] overnight at 4 °C for 12 h and were incubated with the fluorescent antibody (CoraLite594—conjugated goat anti-mouse IgG(H + L), SA00013-3, Proteintech) for 45 min at 37 °C and then with DAPI for 5 min. Finally, the cells were observed under a fluorescence microscope (Axiovert A1, Carl Zeiss AG).

### 2.10. Biological Information Analysis

A homology analysis of the obtained epitopes special to 1E11, 2B3, and 3E5 mAbs was performed using DNASTAR Megalign software (DNASTAR 11.0) to investigate the biological formations of the homology of the HA epitopes among different AIVs. Moreover, the homology modeling of the HA protein of the H9N2 subtype of AIV was performed from the Zhang lab website (https://zhanggroup.org/). Based on the results of homologous modeling, the epitope positions located in the HA protein reacted to the 3D model of HA on Pymol (2.5) software (https://pymol.org) to analyze the mapped spatial characteristics as well as the biological functions of HA epitopes.

### 2.11. Neutralisation Assay

Ninety-six well plates containing monolayers of MDCK cells were infected with 10-fold serial dilutions of H9N2 virus as described in 2.1. After 48 h, the cells were observed for the viral infection by indirect immunofluorescence assay (IFA). The 50% tissue culture infectious dose (TCID_50_) titer of H9N2 virus was calculated by the Reed–Muench method. The mixture of ten-fold serial dilutions of the mAbs and virus suspension containing 200 TCID_50_ of H9N2 was incubated for 1 h at 37 °C, and then added to MDCK cells. After 2 h of adsorption, the viral inoculum was removed, and the cells were cultured in DMEM medium containing 2% FBS.

### 2.12. Haemagglutinin Inhibition (HI) Assay

As previously mentioned, the haemagglutinin (HA) titers of H9N2 were measured [48]. At room temperature, two-fold serial dilutions of the virus were incubated with 0.5% chicken red blood cells (RBCs). The maximum dilution of agglutinated RBCs was used to calculate the HA titer. Four HA units were used in the HI experiment. Four HA units of the virus were reacted with 25 μL of doubly diluted ascites on a 96-well plate for 30 min at room temperature. 0.5% chicken red blood cells were added and incubated for 30 min. HI titers were reported as the reciprocal of the maximum dilution of hemagglutinin that completely inhibited four HA units of virus.

### 2.13. Quantitative Real-Time PCR

Total cellular and supernatant RNA was isolated using the TRIzol method, and the resulting RNA was reverse transcribed into cDNA template using PrimeScriptTM RT Master Mix (RR036A, Takara) and utilized for real-time fluorescence quantitative PCR (RR420A, Takara). The reverse transcription system was 20 μL: total RNA 4 μL, 5×PrimeScript RT Master Mix 4 μL, RNase-free H_2_O 12 μL. The reaction procedure was 37 °C for 15 min. The setup for real-time fluorescence PCR was 20 μL: TB Green premix Ex Taq(2×) 10 μL, Primer-F 0.4 μL, Primer-R 0.4 μL, ROX Reference Dye(50×) 0.4 μL, cDNA 2 μL, DNase/RNase-free H20 6.8 μL. The reaction procedure was: stage1, 95 °C for 30 s; stage 2, 95 °C for 5 s, 60 °C for 30 s, 40 cycle; Dissociation stage, 95 °C for 15 s, 60 °C for 1 min, 95 °C for 15 s. The primers for real-time fluorescence PCR were shown in Table 2.

## 3. Results

### 3.1. Cloning, Expression and Purification of Recombinant HA Protein

The truncated HA target gene was cloned by PCR at a fragment size of 648 bp (Figure 1B), as expected. The positive recombinant plasmid pET-28a-HA was identified by double digestion (Figure 1C), and the plasmid was sequenced to prove sequence integrity in Tsingke Biotechnology Co., Ltd. (Beijing, China). The recombinant HA protein was expressed with a size of 28 KDa, which could react specifically with positive chicken serum kept in our laboratory [13] (Figure 1D). Moreover, the recombinant HA is expressed in inclusion bodies (Figure 1E). Following Ni column affinity chromatography, the recombinant HA protein showed a good purification effect at concentrations from 80 to 250 mM imidazole with the specific target band (Figure 1F).

### 3.2. Screen of Monoclonal Antibody Specific to HA Proteins

**Figure 2:**
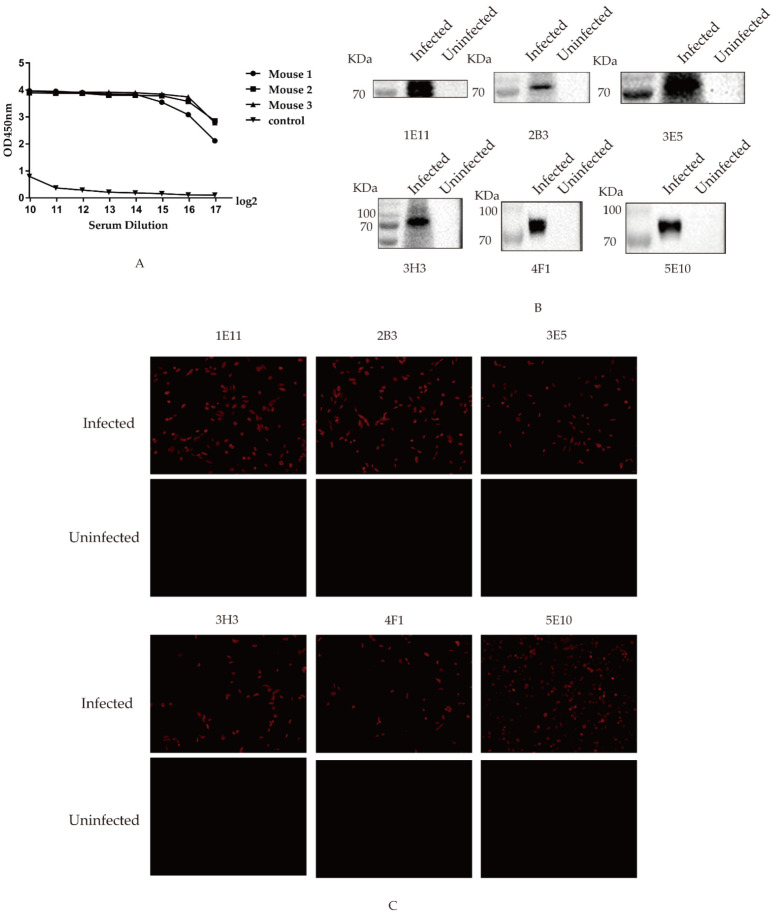
Screening and identification of monoclonal antibodies: (**A**) antibody levels of the immunized mice. At one week after the third immunization, the antibody potency of the immunized mice was detected, and presented as the absorbance value of OD_450 nm_; (**B**) relationship between mAbs 1E11, 2B3, 3E5, 3H3, 4F1, and 5E10 and AIV-infected cells. A549 cells were infected with AIV, and the protein samples were collected at 24 hto detect recognition between HA protein and mAbs by western blotting; and (**C**) reactivity of six mAbs detected by the indirect immunofluorescence. MDCK cells were infected with AIV and incubated with the screened mAbs to detect the reactivity’s mAbs with HA protein of AIV with indirect immunofluorescence. The HA protein was marked in red.

At one week after the third immunization, the serum antibody productions were determined by the indirect ELISA, as shown in Figure 2A. It was observed that the antibody level of No. 3 mouse was the highest among all immunized mice, which was booster immunized with the recombinant protein for hybridoma cell fusion.

At 14 d after cell fusion, the supernatant of the fused cells was detected by the indirect ELISA coated with 2 μg/mL of the recombinant HA protein per well, and six hybridoma cell clones were identified that secreted the specific antibodies to the truncated HA protein and were named 1E11, 2B3, 3E5, 3H3, 4F1, and 5E10, respectively. The type and isotype of monoclonal antibodies were determined by the mouse monoclonal antibody isotype identification kit, shown in Table 3.

To determine whether these mAbs 1E11, 2B3, 3E5, 3H3, 4F1, and 5E10 could recognize the native HA protein, the samples collected from cells infected with H9N2 AIV were detected. Since HA proteins undergo glycosylation modifications [49,50], they are shown to be over 70 KDa in Western blot. The results showed that anti-HA mAbs could recognize the HA protein of AIV at 24 h from cells infected (Figure 2B). Furthermore, the combination of HA mAbs and infected cells shows red light in the figure, and the non-combination of HA monoclonal antibody and uninfected cells shows no fluorescence in the figure. The indirect immunofluorescence verified that all mAbs could bind to cells infected with the virus (Figure 2C), suggesting that these mAbs might have the ability to recognize HA proteins.

### 3.3. Screening of B-Cell Determinants Specific to the Monoclonal Antibody

**Figure 3:**
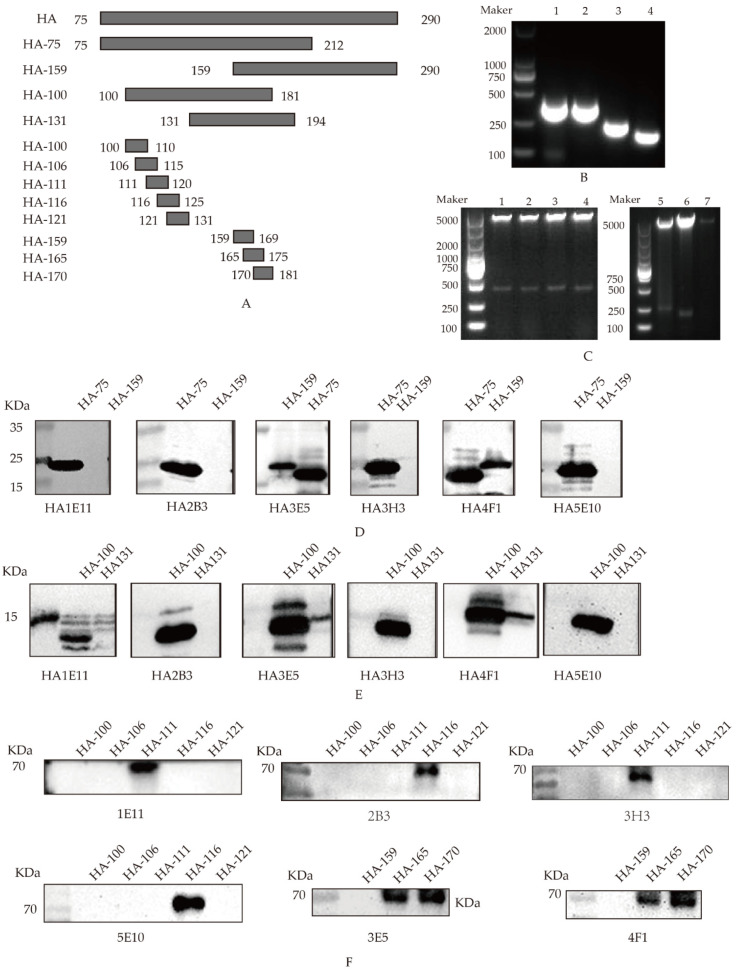
Identification of B cell determinants of mAbs special to HA protein: (**A**) schematic diagram of HA epitope identification; (**B**) PCR amplification. Lane 1, PCR production of HA-75; lane 2, PCR production of HA-159; lane 3, PCR production of HA-100; lane 4, PCR production of HA-131; (**C**) identification of the recombinant plasmids pET-28a-HA-100, pET-28a-HA-159, pET-28a-HA-100, pET-28a-HA-131 with the double enzyme digestion. Lane 1 and lane 2, pET-28a-HA-75; lane 3 and lane 4, pET-28a-HA-159; lane 5, pET-28a-HA-100; lane 6, pET-28a-HA-131; lane 7, pET-28a vector; (**D**) identification of the truncated HA-75 and HA-159 with mAbs following western blot analysis; (**E**) identification of the truncated HA-100 and HA-131 with mAbs following western blot analysis; and (**F**) identification of the coupled peptides with mAbs following western blot analysis.

To confirm the epitopes recognized by anti-HA mAbs, according to the selected HA protein sequence, we constructed four truncated HA fragments and eight synthetic peptides through step-by-step truncation, as shown in Figure 3A. Firstly, two overlapping peptides spanning the truncated HA genes HA-75 (75–212AA) and HA-159 (159–290AA) were cloned into pET-28a(+) (Figure 3B). After the induced expression, the recombinant HA-75 protein was only recognized by 1E11, 2B3, 3H3, 5E10. The recombinant HA-75 and HA-159 proteins were both recognized by 3E5 and 4F1. These results suggested that mAbs 1E11, 2B3, 3H3, and 5E10 could recognize the region 75–157AA in HA protein, while 3E5 and 4F1 might recognize the region 158–212AA in HA protein (Figure 3D).

To further confirm the epitopes recognized by anti-HA mAbs, two overlapping peptides, HA-100 (100–181AA) and HA-131 (131–194AA), spanning the HA genes from 100 to 194 AA, were cloned into prokaryotic expression pET-28a(+) vectors (Figure 3C). The western blotting results showed that the fragment HA-100 protein was recognized by 1E11, 2B3, 3H3 and 5E10 mAbs. The fragment HA-100 and HA-131 proteins were both recognized by 3E5 and 4F1 (Figure 3E). 1E11, 2B3, 3E5 and 4F1 only recognized HA-75 and HA-100, but did not recognize HA-159 and HA131. Their overlapping regions were 100AA to 131AA. These results suggested that mAbs 1E11, 2B3, 3E5 and 4F1 might recognize the region 100AA to 131AA. The region 159AA to 181AA was considered together with the first pair peptide 159–212AA and the result of the second pair peptide (100–181AA and 131–194AA): 131–181AA, then the overlapping peptide was 159–181AA. Therefore, mAbs 3E5 and 4F1 recognized the region 159AA to 181AA in HA protein.

To determine the minimal epitope of the HA protein, five peptides which spanned from 100 to 131AA, ^100^RPSAVNGMCYP^110^, ^106^GMCYPGNVEN^115^, ^111^GNVENLEELR^120^, ^116^LEELRSLFSS^125^, ^121^SLFSSASSYQR^131^ with stepwise pentapeptides, were synthesized and coupled with BSA. Three peptides which spanned from 159 to 181AA, ^159^MRWLTQKNNAY^169^, ^165^KNNAYPIQDAQ^175^, ^170^PIQDAQYTNNRG^181^ with stepwise pentapeptides to hexapeptides, were synthesized and coupled with BSA. The western blotting results showed the peptide ^111^GNVENLEELR^120^ was recognized by 1E11 and 3H3 mAbs, and peptide ^116^LEELRSLFSS^125^ was recognized by 2B3 and 5E10 mAbs, and peptide ^165^KNNAYPIQDAQ^175^ and ^170^PIQDAQYTNNRG^181^ were recognized by 3E5 and 4F1 mAbs (Figure 3F). The reactivity of mAbs with different truncates and peptides were shown in Table 4. Generally, B cell epitope is at least five amino acids. For example, 1E11 recognizes polypeptide HA-111, but does not recognize HA-106 and HA-116. The overlap amino acids between HA-111 and HA-106 is 5AA, and HA-111 and HA-116 have 5AA overlap, indicating that 111–125AA is not an epitope special to 1E11, and 116–120 is not an epitope special to 1E11. Then, one amino acid should be pushed backward, that is, 111AA and 120AA are removed. Therefore, 112–119AA is the epitope that 1E11 may recognize. Similarly, the epitopes position in HA-165 and HA-170 can be deduced in this way. 3E5 can recognize HA-165 and HA-170, and their overlapping fragments are 6 AA, which are the epitopes recognized by 3E5. So, these results suggested that 1E11 and 3H3 mAbs might recognize the epitope located at ^112^NVENLEEL^119^, 2B3 and 5E10 might recognize the epitope located at ^117^EELRSLFS^124^, and 3E5 and 4F1 might recognize the epitope located at ^170^PIQDAQ^175^ in HA protein of H9N2 AIV.

### 3.4. Haemagglutinin Inhibition and Neutralization Assay

To further investigate the characters of mAbs special to HA protein, the haemagglutination inhibitory activity of 1E11, 2B3 and 3E5 mAbs were analyzed by HI assay. The results showed that all three antibodies had haemagglutination inhibitory activity. The HI titers were 2^11^ for 1E11, 2^9^ for 2B3 and 2^11^ for 3E5, indicating that all three epitopes are HA binding sites for red blood cells.

**Figure 4:**
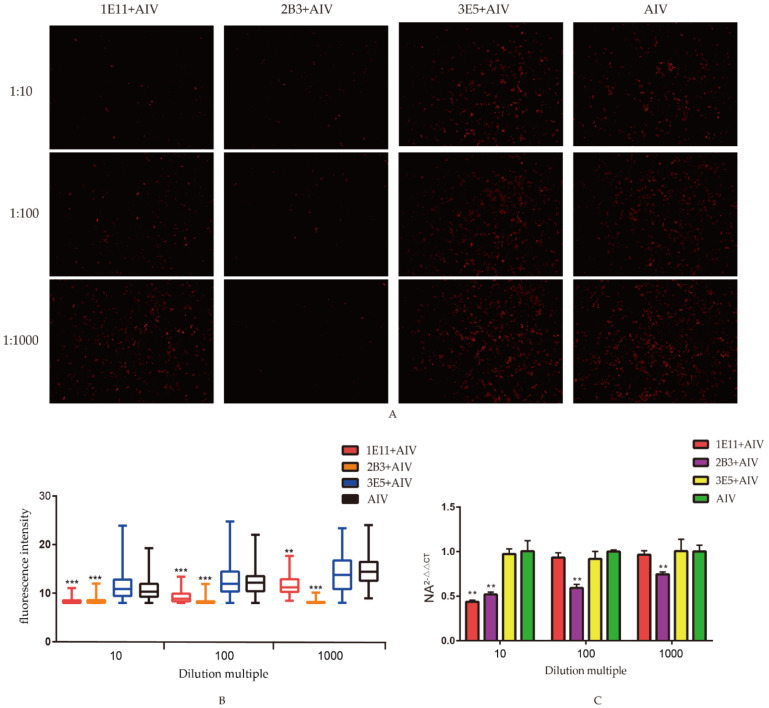
Neutralization assay: (**A**) neutralization effect of the screen mAbs. AIV were co-incubated with three mAbs and infected MDCK cells for 36 h. AIV proliferation in cells was analyzed by the indirect immunofluorescence to analyze the neutralization effect of mAbs; (**B**) the fluorescence intensity in the infected MDCK cells. The software ImageJ was used to quantify the fluorescence intensity of the indirect immunofluorescence images, and the data were represented in Graphpad for the differential analysis; and (**C**) the mRNA levels of NA in MDCK cells. Cells and supernatant total RNA were extracted for real-time fluorescent quantitative PCR, followed by differential analysis using Graphpad. All data were presented as mean ± SD. ** *p* < 0.01, and *** *p* < 0.001.

The results of indirect immunofluorescence and fluorescence intensity analysis showed that the fluorescence intensity of 1E11 was lower than that of the H9N2 virus group, mAbs 1E11 at 10×, 100× and 1000× dilutions, and the differences were significant. 2B3 was lower than that of the H9N2 virus group, 2B3 at 10×, 100× and 1000× dilutions, and the differences were also significant. This suggests that 1E11 and 2B3 mAbs inhibited virus proliferation after co-culture with H9N2 AIV (Figure 4A,B). Fluorescent quantitative PCR results showed that mAb 1E11 inhibited virus proliferation after 10-fold dilution, while 2B3 still inhibited virus proliferation after 1000-fold dilution Figure 4C. In summary, mAbs 1E11 and 2B3 have some neutralizing activity and could inhibit virus proliferation. These results also suggested that the region ^112^NVENLEELRSLFS^124^ recognized by monoclonal antibodies 1E11 and 2B3 might be the epitope which could induce the production of neutralizing antibodies.

### 3.5. The Identified Epitope Is Highly Conserved in AIV Strains

**Figure 5:**
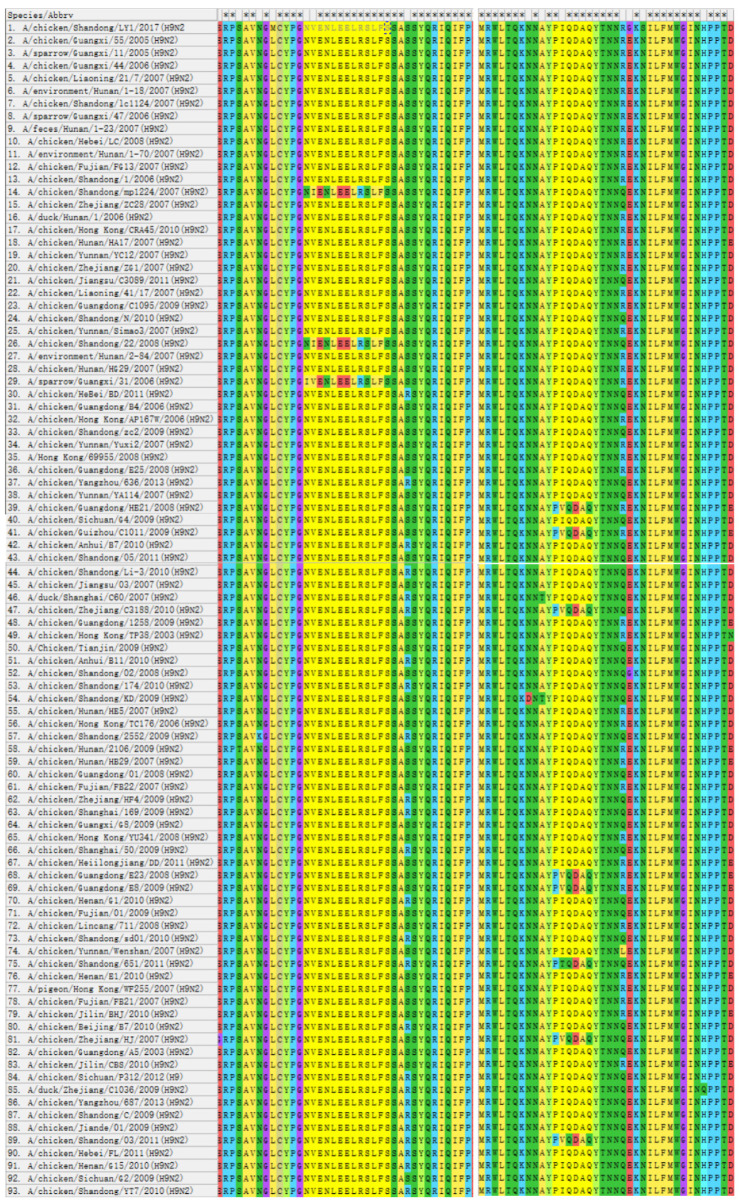
Antigenic epitope motifs were aligned with 93 AIV strains. Homology analysis of the selected sequences was performed by the software MEGA-X in 93 strains. The information for 93 strains H9N2 AIV was showed in Table S1.

This strain, H9N2 (A/chicken/Shandong/LY1/2017), is a member of the avian influenza subtype H9N2 branch that is now most widespread in China, h9.4.2.5 [51,52]. For comparison study, 92 strains of the h9.4.2.5 branch were chosen. In these 93 strains, the N^112^ in epitope ^112^NVENLEEL^119^ was replaced by I^112^ only in H9N2 (A/sparrow/Guangxi/31/2006), H9N2 (A/chicken/Shandong/mp1224/2007) and H9N2 (A/chicken/Shandong/N/2010) with I^113^ replacing V^113^. The H9 subtype AIV epitope ^117^EELRSLFS^124^ is conserved in all 93 strains (Figure 5, Table S1). In the epitope ^170^PIQDAQ^175^, one virus contained T^171^ instead of I^171^, and seven of the 93 H9subtypese AIV had V^171^ instead of I^171^ (Figure 5).

These results indicated that epitope ^117^EELRSLFS^124^ might be extremely conserved in branch h9.4.2.5 and epitope ^112^NVENLEEL^119^ might be likewise moderately conserved, and these two epitopes were substantially conserved neutralizing epitopes. The mAbs targeting these two epitopes might function as a detection and protective antibody for the strain’s h9.4.2.5 branch.

### 3.6. Spatial Location Prediction of Epitope Binding

**Figure 6:**
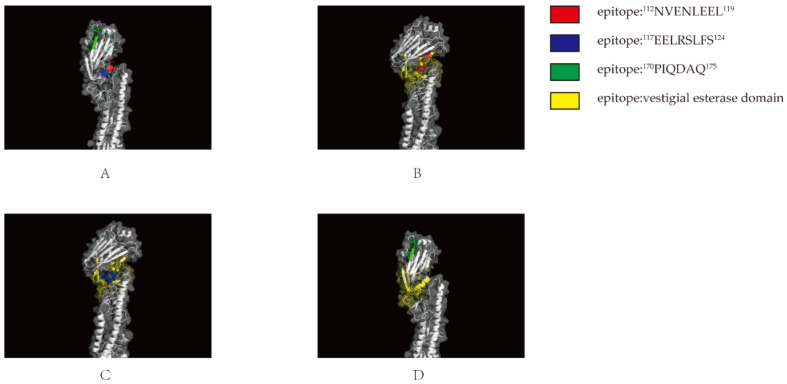
Localization of the identified epitope specific to HA protein: (**A**) epitopes recognized by mAbs 1E11, 2B3 and 3E5. These epitopes were analyzed with “pymol”, in which epitope recognized by 1E11 was marked in red, and epitope recognized by 2B3 was marked in blue, and epitope recognized by 3E5 was marked in green; (**B**) epitopes recognized by 1E11 mAb located in vestigial esterase domain (marked in yellow) and also exposed on the surface of HA protein; (**C**) epitopes recognized by 2B3 mAb located in vestigial esterase domain (marked in yellow) and also exposed on the surface of HA protein; and (**D**) epitopes recognized by 3E5 mAb located in the head of HA and exposed on the surface of HA protein.

The antigenic epitope structure of HA was analyzed using the Zhang lab website (https://zhanggroup.org/, accessed on 1 October 2022) and the software Pymol 2.5 (https://pymol.org, accessed on 1 October 2022). The three epitopes were shown in Figure 6A, and epitope ^112^NVENLEEL^119^ and epitope ^117^EELRSLFS^124^ were in the same α-helix(110-helix), which belong to vestigial esterase (VE) domain, and both epitopes were located on the protein surface (Figure 6B,C). Epitope ^170^PIQDAQ^175^ was located at one β-turn end, and was also located on the protein surface in HA protein (Figure 6D).

## 4. Discussion

H9N2 avian influenza is presently ubiquitous around the world and has been prominent in mainland China since its discovery in 1992 [10,12]. HA gene in H9N2 AIV is one of the most frequently altered genes, making vaccination protection much more challenging. The identification of the B-cell epitope of HA protein will aid in the creation of epitope vaccines as well as in the understanding of the virus’s molecular mechanism, making it simpler to control and eliminate the H9N2 subtype of AIV. In this study, six hybridoma cell lines producing anti HA monoclonal antibodies were identified using the hybridoma technique, and three B-cell epitopes were screened utilizing truncation and peptide scanning procedures. The monoclonal antibodies 1E11 and 2B3 had a partially neutralizing effect and reduced virus multiplication, which also suggested that epitopes ^112^NVENLEEL^119^ and ^117^EELRSLFS^124^ can stimulate the production of neutralizing antibodies.

The antigenic epitopes on HA protein in H9N2 subtype AIV were reported. It was found that six variant sites of 92, 145, 166, 167, 168, and 197 are important in the transmission of H9N2 AIV [42]. Wan et al. [43] identified five new 164, 167, 168, 196 and 207 sites, which can react with the different strains. Additionally, the neutralizing antibodies produced by rSNHA-200 might be employed as a cross-protection vaccination candidate to control and prevent H9N2 AIV infections [44]. Also, some important and neutral sites in the VED region in HA protein of H5N1 subtype AIV were found [45]. However, no neutralizing epitopes targeting the VED region have been identified in H9N2 subtype of AIV.

According to the structural predictions, these two neutralizing epitopes, ^112^NVENLEEL^119^ and ^117^EELRSLFS^124^, are positioned in the same alpha-helix (110-helix) of the vestigial esterase domain(VED), which regulates the pH value of HA during membrane fusion with the host cell [27,53]. The neutralizing activity of the monoclonal antibodies 1E11 and 2B3, which may inhibit the pH-induced conformational changes, prevented viral membrane fusion and inhibited viral invasion. We then performed homology analysis of the three epitopes screened and found that epitope ^117^EELRSLFS^124^ (93/93) was conserved in all 93 strains, and epitopes ^112^NVENLEEL^119^ (90/93) and ^170^PIQDAQ^175^ (85/93) were also relatively conserved. These results suggest that all three epitopes are relatively conserved in the h9.4.2.5 branch, with epitopes ^112^NVENLEEL^119^ and ^117^EELRSLFS^124^ inducing neutralizing antibodies, suggesting that these two epitopes could be candidates for an epitope vaccine against the vestigial esterase (VE) domain of the H9N2 subtype, which might be the first to identify a neutralizing epitope targeting the VED region in H9N2 subtype of AIV. Because the VED region is relatively conservative in the same subtype of AIV, these two epitopes could be candidates for a vaccine against the vestigial esterase domain (VED) of H9N2 subtype AIV.

## 5. Conclusions

In summary, three new B-cell epitopes special to HA protein of H9N2 subtype of AIV were identified in this paper, among which epitopes ^112^NVENLEEL^119^ and ^117^EELRSLFS^124^ induced neutralizing antibodies as well as mAbs 1E11 and 2B3. These two epitopes were found to be located in the 110-helix of the vestigial esterase domain (VED), and it was determined that this region is relatively conserved in the H9N2 subtype, indicating that this region could be used as a broad range of neutralizing antibodies against this subtype. These results provide valuable insights for epitope candidate vaccines against the H9N2 subtype, and can be used as a reference for antibody-based therapies. However, the specific mechanism by which monoclonal antibodies 1E11 and 2B3 neutralize the H9N2 subtype of AIV remains to be investigated.

## Figures and Tables

**Table 1:** Primers of the truncated HA gene for PCR cloning.

Gene Name	Primer Name	Primer Sequence (5′–3′)	Primer Size	*T*m (°C)	Product Size (bp)
HA	HA-F	CGCGGATCCATTGAAGGACTGATCTATGGCAACC	34	73.4	648
	HA-R	CCCAAGCTTACATTGCACTACACAGTTACCACTG	34	73.4	
HA-75	HA-74-F	CGCGGATCCATTGAAGGACTGATCTATGGCAACC	34	74	414
	HA-74-R	ACGCGTCGACCACACTTGTTGTTGTGTCGGTCCTT	35	73	
HA-159	HA-158-F	CGCGGATCCATGAGATGGCTGACTCAAAAGAACA	34	70.8	396
	HA-158-R	ACGCGTCGACACATTGCACTACACAGTTACCACTG	35	69	
HA-100	HA-99-F	CGCGGATCCAGACCATCGGCCGTTAATGGAATGT	34	68.8	246
	HA-99-R	ACGCGTCGACTCCTCTATTATTTGTGTATTGGGCG	35	62.1	
HA-131	HA-130-F	CCGGAATTCCGGATGATAAAGCGAGGGATCAACG	34	69.8	192
	HA-130-R	ACGCGTCGACTCGCACTTGATCCATCATTGCTCTT	35	67	

Note: Horizontal characters are restriction endonuclease sites, as follows: GGATCC, *BamH* I; AAGCTT, *Hind* III; GTCGAC, *Sal* I.

**Table 2:** Primers of real-time fluorescence PCR.

Gene Name	Primer Name	Primer Sequence (5′–3′)	Primer Size	Product Size (bp)
NA	NA-F	TCGGCGACACACCAAGAAATGATG	24	143
	NA-R	ATCCACTTTGATCGTTCGTCCCATC	24	
GAPDH	GAPDH-F	AATTCCACGGCACAGTCAAGGC	22	124
	GAPDH-R	ACAACATACTCAGCACCAGCATCAC	25	

**Table 3:** Monoclonal antibody isotype identification and antibody potency.

mAbs	Antibody Potency of Supernatant	Ascites Potency	Heavy Chain	Light Chain
1E11	1:2000	1:20,000	IgG2b	κ
2B3	1:100,000	1:1,280,000	IgG1	κ
3E5	1:2000	1:40,000	IgG1	κ
3H3	1:20,000	1:640,000	IgG2b	κ
4F1	1:2000	1:20,000	IgG1	κ
5E10	1:10,000	1:80,000	IgG1	κ

**Table 4:** The reactivities of six monoclonal antibodies with the turncated HA protein and the epitope in HA protein.

Vector Truncation	mAbs					
	1E11	2B3	3E5	3H3	4F1	5E10
HA-75	+	+	+	+	+	+
HA-159	-	-	+	-	+	-
HA-100	+	+	+	+	+	+
HA-131	-	-	+	-	+	-
Peptide HA-100	-	-	-	-	-	-
Peptide HA-106	-	-	-	-	-	-
Peptide HA-111	+	-	-	+	-	-
Peptide HA-116	-	+	-	-	-	+
Peptide HA-121	-	-	-	-	-	-
Peptide HA-159	-	-	-	-	-	-
Peptide HA-165	-	-	+	-	+	-
Peptide HA-170	-	-	+	-	+	-

Note: “+” is recognization; “-” is no-recognization.

## Data Availability

The data presented in this study are available upon request from the corresponding author.

## Supplementary Materials

The following supporting information can be downloaded at: https://www.mdpi.com/article/10.3390/v14122739/s1. Table S1: The information for 93 strains H9N2 AIV.
