# Correction: Huang et al. Identification of Unique and Conserved Neutralizing Epitopes of Vestigial Esterase Domain in HA Protein of the H9N2 Subtype of Avian Influenza Virus. *Viruses* 2022, *14*, 2739

**DOI:** 10.3390/v16071069

**Published:** 2024-07-03

**Authors:** Xiangyu Huang, Guihu Yin, Yiqin Cai, Jianing Hu, Jingwen Huang, Qingtao Liu, Xiuli Feng

**Affiliations:** 1 Key Laboratory of Animal Microbiology of China’s Ministry of Agriculture, College of Veterinary Medicine, Nanjing Agricultural University, Nanjing 210095, China; 2020107044@stu.njau.edu.cn (X.H.);; 2 Key Laboratory of Veterinary Biological Engineering and Technology, Ministry of Agriculture, Institute of Veterinary Medicine, Jiangsu Academy of Agricultural Sciences, Nanjing 210014, China; 3 MOE Joint International Research Laboratory of Animal Health and Food Safety, College of Veterinary Medicine, Nanjing Agricultural University, Nanjing 210095, China

## Error in Figure 4A

In the original publication [1], the images of 3E5+AIV at 1:10 and 1:100 in “*Figure 4A*” were duplicated. “Due to an oversight, the manuscript was published with two duplicated images. Given the importance of this information and that the updated image of 3E5+AIV at 1:10 in Figure 4A is provided by the authors, an update of this data is hereby requested”. The corrected “*Figure 4*” appears below. This correction was approved by the Academic Editor. The original publication has also been updated.

**Figure 4:**
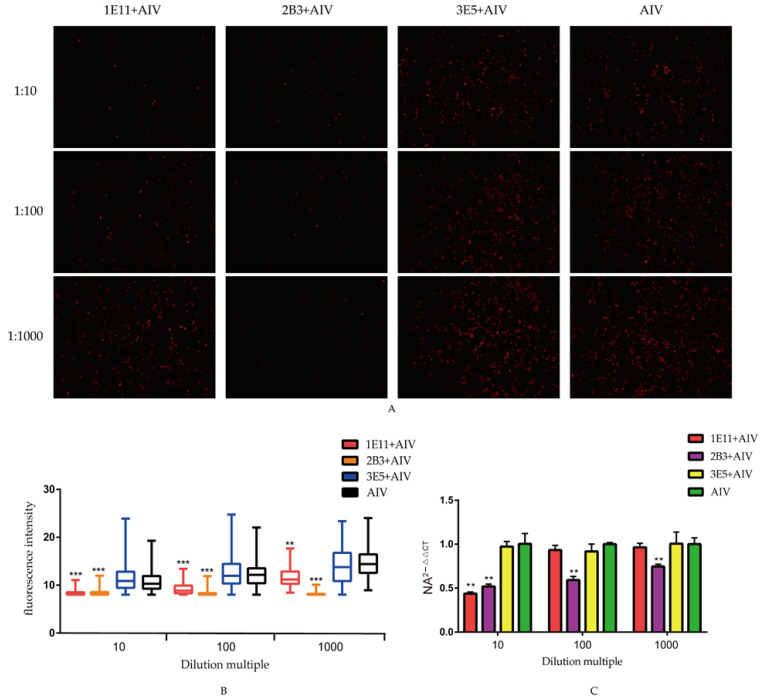
Neutralization assay: (**A**) neutralization effect of the screen mAbs. AIV were co-incubated with three mAbs and infected MDCK cells for 36 h. AIV proliferation in cells was analyzed by the indirect immunofluorescence to analyze the neutralization effect of mAbs; (**B**) the fluorescence intensity in the infected MDCK cells. The software ImageJ was used to quantify the fluorescence intensity of the indirect immunofluorescence images, and the data were represented in Graphpad for the differential analysis; and (**C**) the mRNA levels of NA in MDCK cells. Cells and supernatant total RNA were extracted for real-time fluorescent quantitative PCR, followed by differential analysis using Graphpad. All data were presented as mean ± SD. ** *p* < 0.01, and *** *p* < 0.001.

## References

[1] Huang X., Yin G., Cai Y., Hu J., Huang J., Liu Q., Feng X. (2022). Identification of Unique and Conserved Neutralizing Epitopes of Vestigial Esterase Domain in HA Protein of the H9N2 Subtype of Avian Influenza Virus. Viruses.

